# A case report of metastatic mixed adeno-neuroendocrine carcinoma of the anus presenting as anal pain

**DOI:** 10.1016/j.ijscr.2020.04.065

**Published:** 2020-05-11

**Authors:** Li Xian Lim, Marie Shella De Robles, Robert Duncan Winn, Kimberly Anne Hart

**Affiliations:** a Department of General Surgery, Level 2, Block B, Wollongong Hospital, Loftus Street, Wollongong, NSW 2521, Australia; b Department of Pathology, Level 7, Block C, Wollongong Hospital, 348-352 Crown Street, NSW 2500, Australia

**Keywords:** Case report, Mixed adeno-neuroendocrine carcinoma, Mixed neuroendocrine non-neuroendocrine neoplasm, Small cell carcinoma, Adenocarcinoma

## Abstract

**Introduction:**

Mixed adeno-neuroendocrine carcinoma (MANEC) is a rare disease, and much of the available literature to date has consisted of case reports. A recent systematic review revealed heterogeneity in the data as not all reports documented treatment regimens and course of disease. The recent 2019 WHO update on neuroendocrine carcinoma nomenclature adds to the pre-existing classification system based on biologic activity, to better represent the spectrum of neuroendocrine non-neuroendocrine tumours (Frizziero et al., 2020).

**Presentation of case:**

We present a case of a patient who presented with anal pain, had a wide local excision which on histopathology revealed poorly differentiated MANEC. Despite adjuvant chemotherapy with cisplatin and etoposide as well as pelvic radiotherapy, the patient developed bi-lobar liver metastases within 9 months of initial presentation. The patient succumbed to colonic perforation 10 months after initial presentation.

**Discussion:**

Most patients present with advanced disease with site-specific symptoms, and despite treatment of localised disease, many recur with distant metastasis.

**Conclusion:**

Although rare, this disease is highly aggressive, thus it is hoped that more clinicians can be made aware about its various clinical manifestations and disease course.

## Highlights

• Mixed adeno-neuroendocrine carcinoma can present as benign anal pathology.
• MANECs are aggressive in growth rate and propensity to metastasise.
• Pre-operative staging should be completed even for sub-centimetre lesions.

## 1 Introduction

Mixed adeno-neuroendocrine carcinoma (MANEC) is a rare pathological diagnosis only recently acknowledged by the World Health Organisation in 2010. MANEC is characterised by significant histological heterogeneity. Strictly speaking, each neuroendocrine and non-neuroendocrine component must make up at least 30% of the neoplasm and must be high grade. In 2019, the WHO classification was updated, further subdividing both well and poorly differentiated neoplasms into high and low grades, and introducing the more general terminology – “non-neuroendocrine” [1]. The current conceptual category of mixed neuroendocrine non-neuroendocrine neoplasms, or MiNENs, allows for the possibility that one or both components may be well-differentiated (although in most cases, both tend to be poorly differentiated) [2]. To avoid confusion in this case and in keeping with the original histopathological report, given that both the neuroendocrine and non-neuroendocrine components were found to be high-grade as well as poorly differentiated, the authors decided to maintain consistency in referring to the diagnosis as MANEC. This patient was managed in a major tertiary referral and teaching hospital. In addition, this case has been reported in line with the SCARE criteria [3].

## 2 Presentation of case

**Fig. 1:**
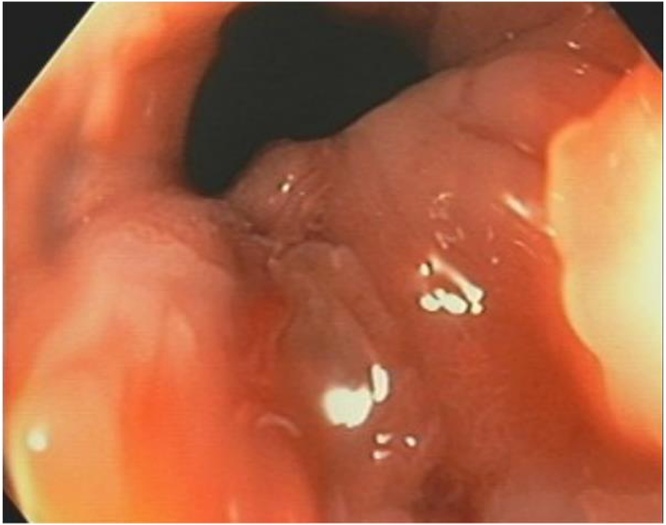
EUA + colonoscopy showed an 8 mm plaque just below dentate on left side of anal canal.

A 48-year-old female self-presented to the Emergency Department complaining of 3 weeks of anal pain which was initially being treated as haemorrhoids. There was no significant family history of malignancy. She was subsequently referred to a colorectal surgeon for persistence of symptoms and described pain in the anus on sitting and pain during the passage of a motion. Examination revealed a sore lump in the anal canal. Colonoscopy and examination of the anus under anaesthesia showed an 8 mm plaque on the dentate line that was biopsied (Fig. 1). Histopathology described a small cell carcinoma, with mucosal glands lined by high grade dysplastic columnar epithelial cells in keeping with small cell carcinoma adenocarcinoma – 20% of neoplastic cells stained for Ki67.

**Fig. 2:**
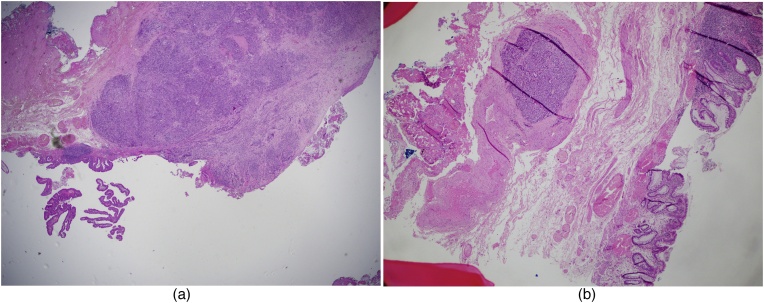
(a) Specimen shows high grade, poorly-differentiated small cell NEC (left) and adenocarcinoma (right) (H&E ×20). (b) Large vessel venous invasion by small cell NEC (left) and glands lined by high grade adenocarcinoma (right) (H&E ×20).

**Fig. 3:**
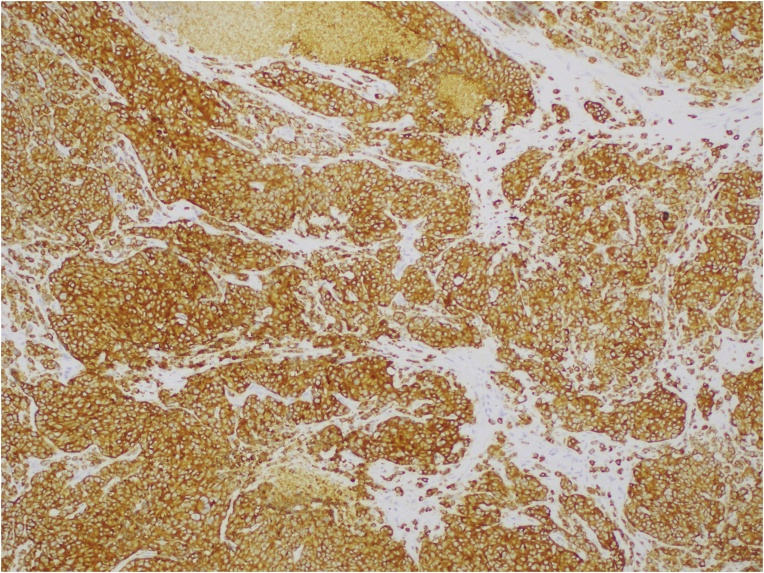
Positive cytoplasmic membrane labelling of synaptophysin in small cell NEC with hyperchromatic nuclei, inconspicuous nucleoli and scant cytoplasm (×100).

The tumour was thought to be very small and locally resectable, and its malignant potential was underestimated. The patient proceeded 13 days later with a wide local excision of the anal lesion with a 15 mm margin. The specimen measured 45 mm in proximal to distal length, 40 mm in width from left lateral to posterior anal canal, and 5 mm in depth. The surface of the tissue showed a rough nodular lesion with ill-defined borders, involving the dentate line and measuring 13 × 10 mm. Final histopathology reported a 1.3 cm poorly differentiated mixed adeno-neuroendocrine carcinoma (MANEC) of the anus (Fig. 2a–b). The neuroendocrine component was a small cell carcinoma which was positive for synaptophysin (Fig. 3) and chromogranin, with a Ki67 index of 80%. It invaded the submucosa and the excision margin was clear. There was however note of lymphovascular invasion. Subsequent staging CT of the chest, abdomen and pelvis fortunately showed no definite evidence of metastatic disease or pelvic or groin lymphadenopathy.

After discussion at the multidisciplinary colorectal meeting, it was recommended that the patient undergo adjuvant chemotherapy given the poor prognosis of most extrapulmonary small cell cancers. The role of radiotherapy is not clear in anal small cell cancer although there is some benefit in small cell cancer of the cervix. The consensus was to give her adjuvant radiotherapy (concurrent with her final cycle of chemotherapy) to minimise relapse, especially in a relatively young and previously healthy individual given that there is no salvage option in this aggressive disease. The patient completed 4 cycles of cisplatin and etoposide chemotherapy as well as pelvic radiotherapy.

**Fig. 4:**
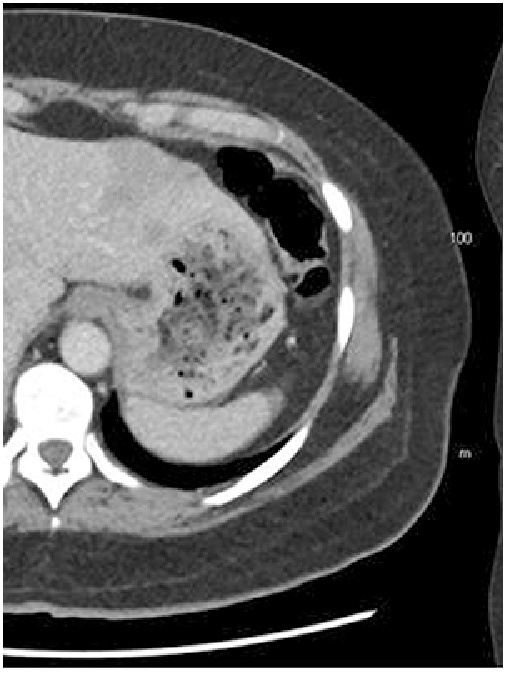
Follow-up CT scan 6 months later demonstrating new liver metastases (axial and coronal views).

Per rectal examination 8 months post initial presentation showed no evidence of local recurrence. CT scan at 9 months post presentation showed no evidence of anal tumour recurrence; however there were multiple bi-lobar liver metastases (Fig. 4). She was commenced on further chemotherapy with FOLFIRI + Avastin as palliative treatment. She continued to struggle with symptoms of profound lethargy, anorexia, weight loss, vague abdominal pains and diarrhoea necessitating several phone calls to the Cancer Care clinic and presentations to the Emergency Department. On one such presentation, she was found to be mildly tachycardic and febrile to 38.9 °C, and was admitted under the Medical Oncology team. She was diagnosed with sepsis secondary to a colonic perforation from diverticular disease with associated *E. coli* bacteraemia after having positive blood cultures and an abdominal CT scan which showed a small amount of localised free intraperitoneal air in the pelvis. She initially improved, with her pain resolving on intravenous antibiotics. Seven days later her abdominal pain returned, and a further CT demonstrated a pelvic collection with free air throughout the peritoneal cavity. The option of a Hartmann’s procedure was discussed. With progressive liver disease, no further chemotherapy options being available and the significant morbidity and mortality associated with the procedure, the patient opted for non-operative management and to be made palliative. She passed away several days later, 10 months after initial presentation.

## 3 Discussion

MANEC is a rare entity. According to the European Union Surveillance of Rare Cancers registry, the incidence was less than 0.1/100,000 persons per year, and there were only 96 cases in the entire continent in 2008 [4]. There is a male preponderance (65.6%) compared to female (22.9%), and the most common primary site was the appendix (60.3%), followed by colon-rectum (14.5%) and stomach (6.7%) [4]. Common biochemical markers suggest that the neuroendocrine and non-neuroendocrine components originate from pluripotent stem cells and undergo divergent differentiation during tumorigenesis [2,5]. An alternative explanation is that as part of tumour progression, the neuroendocrine differentiation develops from the non-neuroendocrine phenotype [1,6].

MANECs are probably underdiagnosed and underestimated, given controversies surrounding definition, tumour sampling errors in biopsies, potential for inadequate immunohistochemical analysis in detecting neuroendocrine components, and an absence of clinical trials studying this disease [1]. Most patients present in an advanced setting with site-specific symptoms such as pain, or constitutional syndrome like weight loss and fatigue, while fewer than 5% of patients present with hormonal syndromes. The latter is postulated to be due to the poorly-differentiated nature of NECs [4]. Workup involves endoscopic assessment or ultrasound-guided percutaneous biopsy, and pre-excision staging with whole-body CT and/or MRI. Octreotide scans can be helpful in neoplasms with low proliferative indices. If distant metastases are suspected, FDG-PET may be useful [4]. Synaptophysin and chromogranin A immunostains are reliable in detecting neuroendocrine differentiation. In a study of 200 surgical samples of MANECs, the Ki67 index of the NEC component was the strongest prognostic marker after adjusting for primary tumour site [6]. Patients with Ki67 ≥ 55% had a shorter median overall survival of 12 months, compared to 40.5 months in those with Ki67 < 55% [6]. Overall survival of advanced MANEC cases is 12–18 months [1].

Due to its rarity, best management practices remain unclear. By convention, small cell cancer in MANEC is managed as small cell lung cancer. Local therapeutic approaches like surgery, radiotherapy and chemoradiation are viable, but despite treatment of localised disease, most recur with distant metastasis [5]. Systemic chemotherapy regimens include platinum-based agents (cisplatin, carboplatin) and topoisomerase inhibitors (etoposide, irinotecan).

## 4 Conclusion

This case highlights the aggressive nature of MANECs both in rate of growth and propensity to metastasise. Anal MANECs can present with minor findings to examination and symptoms similar to benign anal pathologies. Pre-operative staging should be completed before even sub-centimetre lesions are excised.

## Declaration of Competing Interest

None.

## Funding

This paper did not receive any specific grant from funding agencies in the public, commercial, or not-for-profit sectors.

## Ethical approval

The study is exempt from ethical approval at this institution, as per page 4 of the policy directive ‘Research - Ethical & Scientific Review of Human Research in NSW Public Health Organisations’ [PD2010_055].

## Consent

Written informed consent was obtained from the patient’s next-of-kin prior to the submission of this manuscript for publication, including images. Names, initials, age, ethnicity, occupation, and hospital numbers have been omitted. The images used have been similarly de-identified.

## Registration of research studies

NA.

## Guarantor

Robert D. Winn.

## Provenance and peer review

Not commissioned, externally peer-reviewed.

## CRediT authorship contribution statement

**Li Xian Lim:** Writing - original draft, Visualization. **Marie Shella De Robles:** Writing - review & editing. **Robert Duncan Winn:** Conceptualization, Supervision, Writing - review & editing. **Kimberly Anne Hart:** Resources.
